# Drug-Induced Gingival Overgrowth: The Effect of Cyclosporin A and Mycophenolate Mophetil on Human Gingival Fibroblasts

**DOI:** 10.3390/biomedicines8070221

**Published:** 2020-07-17

**Authors:** Dorina Lauritano, Giulia Moreo, Luisa Limongelli, Annalisa Palmieri, Francesco Carinci

**Affiliations:** 1 Department of Medicine and Surgery, Centre of Neuroscience of Milan, University of Milano-Bicocca, 20126 Milan, Italy; moreo.giulia@gmail.com; 2 Interdisciplinary Department of Medicine, University of Bari, 70121 Bari, Italy; luisanna.limongelli@gmail.com; 3 Department of Experimental, Diagnostic and Specialty Medicine, University of Bologna, via Belmoro 8, 40126 Bologna, Italy; plmnls@unife.it; 4 Department of Morphology, Surgery and Experimental Medicine, University of Ferrara, 44121 Ferrara, Italy; crc@unife.it

**Keywords:** mycophenolate mophetil, cyclosporin A, gingival hyperplasia, gingival overgrowth, periodontal disease

## Abstract

Drug-induced gingival overgrowth may occur after a chronic administration of three classes of systemic drugs: Anticonvulsants, immunosuppressants, and calcium channel blockers. This study aimed to investigate how cyclosporin A and mycophenolate mophetil (immunosuppressive drugs) could interfere with human gingival fibroblasts functions, leading to gingival enlargement. Human gingival fibroblasts derived from the tissue of a 60-year-old female were cultured in a DMEME medium. A stock solution with 1 mg/mL of mycophenolate and 1 mg/mL of cyclosporine were prepared and dissolved in a DMEM medium to prepare a serial dilution at the concentrations of 5000, 2000, 1000, 500, and 100 ng/mL, for both treatments. Cell viability was measured using the PrestoBlue™ Reagent Protocol. Quantitative real-time RT-PCR was performed in order to analyze the expression of 57 genes coding for gingival fibroblasts “Extracellular Matrix and Adhesion Molecules”. Mycophenolate and cyclosporine had no effect on fibroblast cell viability at 1000 ng/mL. Both the treatments showed similar effects on the expression profiling of treated cells: Downregulation of most extracellular matrix metalloproteases genes (*MMP8*, *MMP11*, *MMP15*, *MMP16*, *MMP24*) was assessed, while *CDH1*, *ITGA2*, *ITGA7*, *LAMB3*, *MMP12*, and *MMP13* were recorded to be upregulated in fibroblasts treated with immunosuppressive drugs. It has been demonstrated that gingival overgrowth can be caused by the chronic administration of cyclosporin A and mycophenolate mophetil. However, given the contrasting data of literature, further investigations are needed, making clear the possible effects of immunosuppressive drugs on fibroblasts.

## 1. Introduction

Gingival fibromatosis (also called gingival overgrowth or hyperplasia) can be defined as a pathological diffused or local growth of marginal and attached gingiva or interdental papilla [1]. Gingival overgrowth (GO) may cause severe consequences for the dento-maxillary apparatus: Inflammatory reaction may aggravate the periodontal condition, leading to tooth loss, while speech capacity, chewing, and aesthetic may be altered [2,3]. The ethiopathogenetic mechanisms may derive from idiopathic, hereditary, or drug-induced forms [1]. Drug-induced GO is a side-effect, which may occur after the administration of certain systemic drugs, whose target organ is not the gingival tissue [4]. According to the literature, GO may be related to more than 20 drugs [5,6,7] and, in particular, it could manifest itself as a result of chronic usage of three main classes of drugs: Anticonvulsants, immunosuppressants, and calcium channel blockers [8]. A study by Hatahira et al. (2017) [9] reported that more than 70% of GO incidence was associated with cyclosporine (immunosuppressant) administration, while nifedipine (calcium channel blocker) and phenytoin (anticonvulsant) administration cause GO in 6–15% and 50% of individuals, respectively. The American Academy of Periodontology (2004) recorded a prevalence rate related to nifedipine, cyclosporine, and phenytoin of 6–15%, 25–30%, and 50%, respectively. A connective tissue response, characterized by an excessive accumulation of extracellular matrix proteins or amorphous ground substance, seems to be responsible for the increase of gingival tissue volume [10,11]. Possible risk factors for a drug-induced GO onset are: Poor plaque control, male gender (with a risk three times higher than the female), age (with inverse correlation), and genetic predisposition [10,12]. Cyclosporine A (CsA) provides an immunosuppressive effect and it is widely administrated in case of organ transplantation, in order to prevent acute or chronic rejection and in patients with autoimmune diseases. Mycophenolate (Mf) is used in many different immunosuppressive protocols, since it was demonstrated that it has a manageable toxicity: The study by van Gelder et al. showed that Mf, added to CsA in an immunosuppressive protocol, reduced the incidence of acute rejection after renal transplantation from 40–45% to 20–25% compared to placebo or azathioprine-based therapies [13,14]. The action of both drugs is directed to the T-cell activation cascade. The inhibition of transcription performed by CsA is made possible thanks to the formation of a heterodimeric complex (consisting of the drug and its cytoplasmic receptor protein: Cyclophilin), which binds calcineurin and inhibits its phosphatase activity; in this way the expression of nuclear regulatory proteins and T-cell activation genes are inhibited. Mf develops its function of nucleotide synthesis inhibitor, stopping the action of inosine monophosphate dehydrogenase and thus preventing the biosynthesis of guanosine and deoxyguanosine; this cascade of event leads to a selective inhibition of T- and B-cell proliferation [15]. Although the association between CsA and gingival hyperplasia has been widely demonstrated [4,16], the role of Mf in GO remains unclear, since the number of the researches in this regard is limited: Trandafir et al. [17] reported the case of a 29 year-old male patient, receiving immunosuppressive therapy with CsA and Mf, who developed gingival enlargement; furthermore, a study in 2010 compared two immunosuppressive protocols, which included cyclosporine and prednisolone with Mf or azathioprine, respectively, reporting no difference in terms of gingival overgrowth [18]. However, the study by de la Rosa García [19] supported the theory according to which Mf may present a protective effect against GO.

### 1.1. Role of Cyclosporine A and Mycophenolate Mophetil on Gingival Hyperplasia

GO becomes clinically observable 1 or 3 months after the start of the immunosuppressive therapy and reaches a plateau phase at 9 to 12 months: It manifests initially on the interdental papillae as a localized nodular enlargement, later extending to the dental crown (from an horizontal to a vertical growth) and it is more frequent in the vestibular side of the maxillary and mandibular frontal regions [12,20,21]. It has been demonstrated that, unlike the anticonvulsants and calcium channel blockers-induced gingival hyperplasia, GO associated with the immunosuppressive therapy is characterized by a high inflammation level with a low fibrosis: In particular, CsA leads to an exaggerated innate immune response, targeting cyclophilin and inhibiting T-cell production of interleukin-2 and it provides an anti-fibrotic effect on collagen biosynthesis and deposition [22]. From the histological point of view, the enlargement of the gingiva caused by CsA and Mf could be defined as a connective tissue disorder, marked by incremented interstitial collagen deposition and extracellular matrix proteins production, plasma cell infiltration, increased inflammatory response (increased number of inflammatory cells, such as macrophages), and altered vascularisation [17,23,24]. As reported in the literature, immunosuppressive drugs may interfere with the gingival fibroblasts (GFs) function, altering the transcription of several cytokines, such as transforming growth factor-β1 (*TGF-β1*), interleukin-6 and -8 (*IL-6*, *IL-8*), interleukin-1β (*IL-1β*), and fibroblast growth factor (*FGF*) [5,25,26]. The proliferation of human gingival fibroblasts (HGF) could be stimulated by the long term administration of immunosuppressive drugs [16,27,28,29]; however, some researches highlighted that the effect of CsA on gingival fibroblasts depends on its dose: A long-term exposure to a low dose of the drug (≤200 ng/mL) does not have any influence on the viability and proliferation of these cells, parameters which are inhibited by higher doses of CsA (400-800 ng/mL) [30,31]. *IL-6* is recorded to be highly expressed by the gingival connective tissue fibroblasts in the case of immunosuppressive therapy induced GO, increasing the synthesis of collagen and glycosaminoglycan [10]. Gong et al. [32] recorded higher gingival crevicular fluid *IL-1β* levels in CsA induced GO, which is responsible for the upregulation of the *IL-6* secretion in gingival fibroblasts. Wu et al. [33] found out that the higher expression of *TGF-β1*, which has the capacity to enhance collagen production and to decrease collagen degradation, may promote the profibrotic activity of CsA. The same study demonstrated that CsA also increases the synthesis of the connective tissue growth factor (*CTGF/CCN_2_*), which induces the profibrotic effect of *TGF-β1*.

### 1.2. Objective

An increase in gingival tissue following the administration of anticonvulsants, immunosuppressants, and calcium channel blockers drugs are characterized by fibroblasts hyperproliferation of the connective tissue with a deposition of the extracellular matrix. In addition, cell proliferation involves epithelial-mesenchymal transition resulting in changes in the expression of adhesion molecules.

In this study, the authors investigated how cyclosporin A and michophenolate mophetil could affect human gingival fibroblasts, leading to the onset of gingival overgrowth. In particular, the cell viability and the expression of 57 genes coding for gingival fibroblasts “Extracellular Matrix and Adhesion Molecules” were assessed.

## 2. Experimental Section

### 2.1. Primary Human Fibroblast Cells Culture

Fibroblasts at the second replication step, originating from the gingiva of a 60-year-old woman, were purchased from ATCC^®^ Cell Lines. The fibroblasts were maintained in a humidified atmosphere of 5% CO_2_, at 37 °C and replicated in a DMEM medium added with 10% fetal calf serum (FBS), antibiotics, and amino acids (Sigma-Aldrich, Inc., St Louis, MO, USA).

### 2.2. Cell Viability Test

Human gingival fibroblasts were seeded into 96-well plates at a density of 10^4^ cells per well containing 100 µL of a cell culture medium and incubated for 24 h to allow cell adherence.

A stock solution with 1 mg/mL of mycophenolate and 1 mg/mL of cyclosporine were prepared and dissolved in a DMEM medium to prepare the serial dilution at concentrations of 5000, 2000, 1000, 500, and 100 ng/mL, for both treatments.

A set of wells were treated with mycophenolate, three wells for each concentration. Another set of wells were treated with cyclosporine. The cell culture medium alone was used as a negative control.

After 24 h of incubation, cell viability was measured using the PrestoBlue™ Reagent Protocol (Invitrogen, Carlsbad, CA, USA) according to the manufacturer’s instructions, as previously described [5].

### 2.3. Cell Treatment and RNA Isolation

Fibroblasts seeded at the density of 1.0 × 10^5^ cells/mL were cultivated for 16 h with a serum-free medium in order to normalize the gene expression of the cells.

Subsequently, the cells were treated with cyclosporine and mycophenolate. Each solution was prepared dissolving 1000 ng/mL of the treatment in a DMEM medium containing 2% of FBS, antibiotics, and amino acids. At the end of the treatment, which lasted 24 h, the cells were detached by trypsin and the RNA was isolated using the GenElute mammalian total RNA purification miniprep kit (Sigma-Aldrich), according to the manufacturer’s instructions.

### 2.4. Reverse Transcription and Quantitative Real-Time RT-PCR

The fibroblast cDNA was synthesized starting from 500 ng of total RNA using the PrimeScript RT Master Mix kit (Takara Bio Inc., Kusatsu, Shiga Prefecture, Japan) and subsequently used to amplify the genes belonging to the “Extracellular Matrix and Adhesion Molecules” pathway using a custom primer (Sigma-Aldrich, Inc., St Louis, MO, USA).

The genes were selected on the basis of superarray platforms (SAB Biosciences, Frederick, MD, USA) that analyze specific pathways of interest.

The amplification was conducted on a quantitative real-time PCR using the ViiA™ 7 System (Applied Biosystems, Foster City, CA, USA). The CDNA synthesis and amplification protocol were previously described by Lauritano et al. 2019 [34].

### 2.5. Statistical Analysis

The gene expression analysis was carried out using the delta/delta Ct method [35] using *RPL13* as a reference gene. Each fold change was obtained by comparing the gene expression of the treated cells compared to the control ones.

## 3. Results

The cell viability test conducted on cells served to establish the most suitable concentration for an in vitro treatment such as to determine changes in gene expression without however killing the cells. The PrestoBlue™ assay determined that this concentration for both the treatments was 1000 ng/mL.

**Figure 1:**
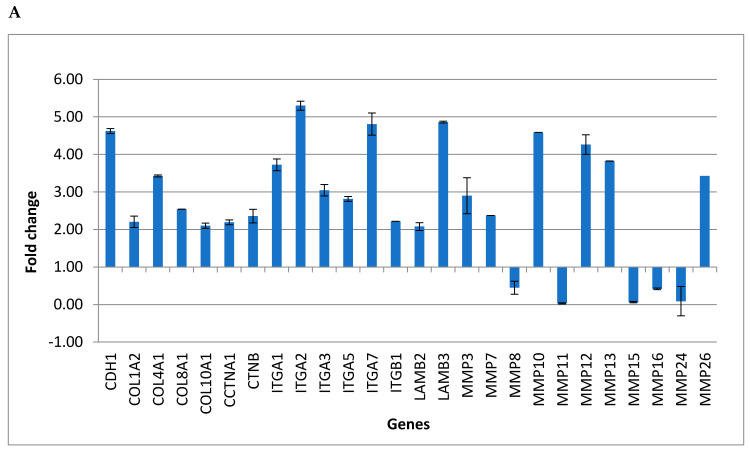

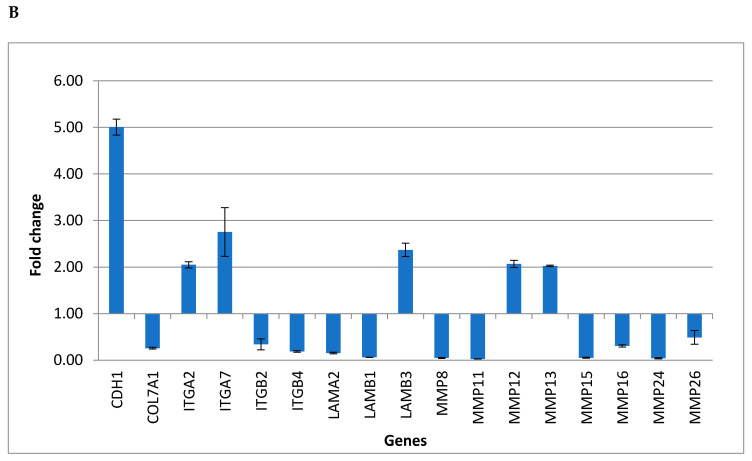
Significant expression levels of the genes up- and downregulated in fibroblast cells treated with mycophenolate (**A**) and cyclosporine (**B**) [34].

In order to evaluate the effects induced by cyclosporine and mycophenolate, 57 genes belonging to the “Extracellular Matrix and Adhesion Molecules” pathway were amplified in fibroblasts after 24 h of treatment. Table 1 and Table 2 show the gene expression values as a Fold Change following the treatment with mycophenolate and cyclosporine, respectively. The bold values represent the statistically significant results (fold change ≥ 2 and *p*-value ≤ 0.05 for upregulated genes, and fold change ≤ 0.5 and *p*-value ≤ 0.05 for significantly downregulated genes) that were collected in Table 3 and Table 4. Following the treatment for 24 h, both substances induced overexpression of the same genes in fibroblasts, including the adhesion molecules *CDH1* and genes involved in the deposition of the extracellular matrix such as *ITGA2*, *ITGA7*, *LAMB3*, *MMP12*, and *MMP13*. Among the genes significantly downregulated by both treatments there are the extracellular matrix metalloproteases *MMP8*, *MMP11*, *MMP15*, *MMP16*, *MMP24*. Figure 1A,B represents significantly expression levels of the genes up- and downregulated in fibroblast cells treated with mycophenolate and cyclosporine, respectively. In order to confirm the expression results obtained by the real-time PCR, it was decided to measure the protein levels of CDH1 by the enzyme immunoassay test (ELISA). The ELISA test confirmed the real-time PCR results showing an increase in CDH1 equal to 5.13-fold ± 0.16 for the mycophenolate treatment and 5.05-fold ± 0.20 for the cyclosporine treatment.

Authors decided to evaluate the *CDH1* expression because it is one of the genes that showed the greatest fold change. Furthermore, adhesion proteins such as CDH1 being expressed on the cell surface are easily quantifiable with immunoenzymatic methods such as ELISA.

## 4. Discussion

Our study aimed at establishing how the immunosuppressive therapy with cyclosporine A and mycophenolate mophetil could affect the viability of human gingival fibroblasts and how they may interfere with fibroblasts gene expression, contributing to the development of gingival hyperplasia. The fibroblasts represent the main cellular category in the connective tissue and they carry out very important functions, along with collagen precursor (tropocollagen) and extra-cellular matrix (ECM) precursor secretion and the connective tissue structural integrity preservation. Fibroblasts also have the capacity to regulate the interstitial fluid volume and pressure and they play a role in wound healing. These cells also produce matrix metalloproteinases (MMP) and their inhibitors (tissue inhibitors metalloproteinases, TIMP), whose role is to regulate the extracellular degradation of ECM [36]. Along with the role of supporting the connective tissue structure, fibroblasts can produce and respond to cytokines (IL-6, IL-8), chemokines, and growth factors (TGF-β1) [37]. As the literature reports, gingival hyperplasia induced by immunosuppressive drugs may be the result of the interference with fibroblast proliferation and collagen, ECM molecules, cytokines and growth factors regulation, synthesis, and processing [38]. In this research, fibroblasts treated with both Mf and CsA solutions at the concentration of 1000 ng/mL for 24 h, showed a downregulation of extracellular matrix metalloproteases (*MMP8*, *MMP11*, *MMP15*, *MMP16*, *MMP24*). Since the family of MMP, including more than twenty enzymes, is responsible for the catabolic turnover of extracellular matrix of the connective tissue, the alteration of the activation of these enzymes may lead to connective tissues accumulation [39,40]. Among the inhibited extracellular matrix metalloproteases there was MMP8. MMP8, also known as neutrophil collagenase, can be produced by fibroblasts, epithelial and endothelial cells, macrophages and neutrophils; its main function is the degradation of type I, II, and III collagens and it emerged to be an anti-tumorigenic and anti-metastatic molecule [41,42]. Another downregulated MMP was demonstrated to be MMP11; during normal and pathological conditions it is mostly expressed by fibroblasts later than the other MMPs, indicating that it is involved in downstream tissue remodelling processes; this MMP is secreted under an active form (contrary to the other enzymes of the same family), it has anti-apoptotic function but it does not have the capacity of degrading any major ECM component [43]. On the contrary, our study demonstrated that cell adhesion genes (*CDH1*) received an upregulation during the fibroblasts treatment with both immunosuppressive drugs. The *CDH1* gene encodes for the E-cadherin (or epithelial cadherin), which is a protein that contributes to sticking epithelial cells to each other. Another function of E-cadherin is that of transmitting chemical signals within cells and it acts as a tumor suppressor protein [44]. Data obtained by the enzyme linked immunoassay (ELISA) performed in our study demonstrated higher levels of CDH1 in fibroblasts treated with CsA and Mf than in untreated cells. Contrary to what emerged in our research, the results recorded by Tu et al. [45] highlighted that CsA could cause a decrease in the production of E-cadherin; other authors supported the same theory: The CsA treatment downregulates the expression of E-cadherin in gingival tissue, an event that compromises basal membrane structure and increases the interaction between epithelial and connective tissue, triggering the fibrotic process [46,47]. The 24-h treatment is probably too short to induce a significant change in the expression of the *CDH1* gene. Longer time-points should be tested in order to verify the behaviour of this adhesion molecule following treatments with cyclosporin and mycophenolate. The literature reported that the administration of CsA induces the phenomenon of epithelial-mesenchymal transition (EMT) in gingival tissue: EMT is a process in which epithelial cell-cell contacts are weakened, acquiring typical mesenchymal cells characteristics [48]. The reduction of E-cadherin production represents a common sign of EMT. ECM degradation can be performed by metalloproteinase and also through fibroblast phagocytosis, which is regulated by integrins [49,50]. Abnormalities in the expression of the *ITGA2* gene, encoding for α2-integrin may lead to altered adherence of fibroblasts to collagen, inhibiting phagocytosis [51]. An upregulation of the *ITGA2* gene of treated fibroblasts was observed in our study. Zhou et al. confirmed the higher expression of this gene in human gingival fibroblasts treated with CsA, but, on the contrary, Liu et al. [52] found that CsA-treated fibroblasts had a downregulation of *ITGA2*, compared to the controls.

The results obtained in vitro are indicative of a possible mechanism of action of immunosuppressive drugs on cells. However, these models need further study concerning both the treatment timing and the choice of models that can mimic conditions in vivo as much as possible.

## 5. Conclusions

A chronic administration of cyclosporin A and mycophenolate mophetil was proved to lead to gingival overgrowth. Connective tissue accumulation (caused by inhibition of metalloproteases), upregulation of *CDH1* (encoding for E-cadherin), *ITGA2*, *ITGA7*, *LAMB3*, *MMP12*, and *MMP13* genes were demonstrated to characterize gingival enlargement induced by immunosuppressive therapy. However, contrasting results reported in the literature require the need for further investigation, in order to clarify the mechanisms involved in this pathologic process.

## Figures and Tables

**Table 1:** Gene expression profile of 57 genes belonging to the “Extracellular Matrix and Adhesion Molecules” pathway analyzed using real-time PCR after 24 h of treatment with mycophenolate.

Gene	Fold Change	Gene Function
*CD44*	1.56	Cell-Cell Adhesion (MIM:107269)
*CDH1*	**4.62**	Cell-Cell Adhesion (MIM:192090)
*COL1A2*	**2.20**	Collagens and Extracellular Matrix Structural constituent (MIM:192090)
*COL2A1*	1.71	Collagens and Extracellular Matrix Structural constituent (MIM:120140)
*COL3A1*	1.71	Collagens and Extracellular Matrix Structural constituent (MIM:120180)
*COL4A1*	**3.43**	Collagens and Extracellular Matrix Structural constituent (MIM:120130)
*COL5A1*	1.31	Collagens and Extracellular Matrix Structural constituent (MIM:120215)
*COL6A1*	1.69	Collagens and Extracellular Matrix Structural constituent (MIM:120220)
*COL7A1*	0.74	Collagens and Extracellular Matrix Structural constituent (MIM:120120)
*COL8A1*	**2.54**	Collagens and Extracellular Matrix Structural constituent (MIM:120251)
*COL9A1*	1.42	Collagens and Extracellular Matrix Structural constituent (MIM:120210)
*COL10A1*	**2.10**	Collagens and Extracellular Matrix Structural constituent (MIM:120110)
*COL11A1*	1.95	Collagens and Extracellular Matrix Structural constituent (MIM:120280)
*CTNNA1*	**2.19**	Cell Adhesion Molecule (MIM:116805)
*CTNNB1*	**2.36**	Cell Adhesion Molecule (MIM:116806)
*CTNND2*	0.95	Cell Adhesion Molecule (MIM:604275)
*FN1*	1.03	Cell Adhesion Molecule (MIM:135600)
*HAS1*	1.86	Transmembrane Receptor (MIM:601463)
*ILF3*	1.73	Transmembrane Receptor (MIM:603182)
*ITGA1*	**3.72**	Transmembrane Receptor (MIM:192968)
*ITGA2*	**5.30**	Transmembrane Receptor (MIM:192974)
*ITGA3*	**3.04**	Transmembrane Receptor (MIM:605025)
*ITGA4*	1.71	Transmembrane Receptor (MIM:192975)
*ITGA5*	**2.82**	Transmembrane Receptor (MIM:135620)
*ITGA6*	1.84	Transmembrane Receptor (MIM:147556)
*ITGA7*	**4.81**	Transmembrane Receptor (MIM:600536)
*ITGA8*	1.82	Transmembrane Receptor (MIM:604063)
*ITGB1*	**2.22**	Transmembrane Receptor (MIM:135630)
*ITGB2*	0.91	Transmembrane Receptor (MIM:600065)
*ITGB4*	0.59	Transmembrane Receptor (MIM:147557)
*ITGB5*	1.46	Transmembrane Receptor (MIM:147561)
*LAMA1*	1.30	Basement Membrane Constituent (MIM:150320)
*LAMA2*	0.12	Basement Membrane Constituent (MIM:156225)
*LAMA3*	1.63	Basement Membrane Constituent (MIM:600805)
*LAMB1*	0.50	Basement Membrane Constituent (MIM:150240)
*LAMB2*	**2.08**	Basement Membrane Constituent (MIM:150325)
*LAMB3*	**4.86**	Basement Membrane Constituent (MIM:150310)
*MMP2*	1.61	Extracellular Matrix Protease (MIM:120360)
*MMP3*	**2.90**	Extracellular Matrix Protease (MIM:185250)
*MMP7*	**2.37**	Extracellular Matrix Protease (MIM:178990)
*MMP8*	**0.45**	Extracellular Matrix Protease (MIM:120355)
*MMP9*	1.43	Extracellular Matrix Protease (MIM:120361)
*MMP10*	**4.59**	Extracellular Matrix Protease (MIM:185260)
*MMP11*	**0.03**	Extracellular Matrix Protease (MIM:185261)
*MMP12*	**4.26**	Extracellular Matrix Protease (MIM:601046)
*MMP13*	**3.82**	Extracellular Matrix Protease (MIM:600108)
*MMP14*	1.20	Extracellular Matrix Protease (MIM:600754)
*MMP15*	**0.06**	Extracellular Matrix Protease (MIM:602261)
*MMP16*	**0.42**	Extracellular Matrix Protease (MIM:602262)
*MMP24*	**0.09**	Extracellular Matrix Protease (MIM:604871)
*MMP26*	**3.43**	Extracellular Matrix Protease (MIM:605470)
*TGFB1*	1.51	TGF-β Signaling (MIM:190180)
*TGFB2*	1.25	TGF-β Signaling (MIM:190220)
*TGFB3*	1.05	TGF-β Signaling (MIM:190230)
*TIMP1*	1.09	Extracellular Matrix Protease Inhibitor (MIM:305370)
*VCAN*	1.07	Cell Adhesion Molecule (MIM:118661)
*RPL13*	1.00	Housekeeping gene (MIM:113703)

The bold values represent the statistically significant results (fold change ≥ 2 and *p*-value ≤ 0.05 for upregulated genes, and fold change ≤ 0.5 and *p*-value ≤ 0.05 for significantly downregulated genes).

**Table 2:** Gene expression profile of 57 genes belonging to the “Extracellular Matrix and Adhesion Molecules” pathway analyzed using real-time PCR after 24 h of treatment with cyclosporine [34].

Gene	Fold Change	Gene Function
*CD44*	0.74	Cell-Cell Adhesion (MIM:107269)
*CDH1*	**5.01**	Cell-Cell Adhesion (MIM:192090)
*COL1A2*	0.92	Collagens and Extracellular Matrix Structural constituent (MIM:192090)
*COL2A1*	0.62	Collagens and Extracellular Matrix Structural constituent (MIM:120140)
*COL3A1*	0.85	Collagens and Extracellular Matrix Structural constituent (MIM:120180)
*COL4A1*	1.73	Collagens and Extracellular Matrix Structural constituent (MIM:120130)
*COL5A1*	0.62	Collagens and Extracellular Matrix Structural constituent (MIM:120215)
*COL6A1*	0.82	Collagens and Extracellular Matrix Structural constituent (MIM:120220)
*COL7A1*	**0.26**	Collagens and Extracellular Matrix Structural constituent (MIM:120120)
*COL8A1*	0.85	Collagens and Extracellular Matrix Structural constituent (MIM:120251)
*COL9A1*	0.91	Collagens and Extracellular Matrix Structural constituent (MIM:120210)
*COL10A1*	0.97	Collagens and Extracellular Matrix Structural constituent (MIM:120110)
*COL11A1*	0.85	Collagens and Extracellular Matrix Structural constituent (MIM:120280)
*CCTNA1*	1.25	Cell Adhesion Molecule (MIM:116805)
*CTNB*	1.27	Cell Adhesion Molecule (MIM:116806)
*CTNND2*	0.96	Cell Adhesion Molecule (MIM:604275)
*FN1*	0.56	Cell Adhesion Molecule (MIM:135600)
*HAS1*	0.97	Transmembrane Receptor (MIM:601463)
*ILF3*	0.93	Transmembrane Receptor (MIM:603182)
*ITGA1*	1.31	Transmembrane Receptor (MIM:192968)
*ITGA2*	**2.05**	Transmembrane Receptor (MIM:192974)
*ITGA3*	1.47	Transmembrane Receptor (MIM:605025)
*ITGA4*	0.99	Transmembrane Receptor (MIM:192975)
*ITGA5*	1.51	Transmembrane Receptor (MIM:135620)
*ITGA6*	1.08	Transmembrane Receptor (MIM:147556)
*ITGA7*	**2.75**	Transmembrane Receptor (MIM:600536)
*ITGA8*	0.77	Transmembrane Receptor (MIM:604063)
*ITGB1*	1.03	Transmembrane Receptor (MIM:135630)
*ITGB2*	**0.34**	Transmembrane Receptor (MIM:600065)
*ITGB4*	**0.19**	Transmembrane Receptor (MIM:147557)
*ITGB5*	0.63	Transmembrane Receptor (MIM:147561)
*LAMA1*	0.71	Basement Membrane Constituent (MIM:150320)
*LAMA2*	**0.15**	Basement Membrane Constituent (MIM:156225)
*LAMA3*	0.89	Basement Membrane Constituent (MIM:600805)
*LAMB1*	**0.06**	Basement Membrane Constituent (MIM:150240)
*LAMB2*	1.08	Basement Membrane Constituent (MIM:150325)
*LAMB3*	**2.37**	Basement Membrane Constituent (MIM:150310)
*MMP2*	0.77	Extracellular Matrix Protease (MIM:120360)
*MMP3*	1.05	Extracellular Matrix Protease (MIM:185250)
*MMP7*	0.78	Extracellular Matrix Protease (MIM:178990)
*MMP8*	**0.05**	Extracellular Matrix Protease (MIM:120355)
*MMP9*	0.92	Extracellular Matrix Protease (MIM:120361)
*MMP10*	1.70	Extracellular Matrix Protease (MIM:185260)
*MMP11*	**0.03**	Extracellular Matrix Protease (MIM:185261)
*MMP12*	**2.07**	Extracellular Matrix Protease (MIM:601046)
*MMP13*	**2.03**	Extracellular Matrix Protease (MIM:600108)
*MMP14*	0.98	Extracellular Matrix Protease (MIM:600754)
*MMP15*	**0.05**	Extracellular Matrix Protease (MIM:602261)
*MMP16*	**0.31**	Extracellular Matrix Protease (MIM:602262)
*MMP24*	**0.04**	Extracellular Matrix Protease (MIM:604871)
*MMP26*	**0.49**	Extracellular Matrix Protease (MIM:605470)
*TGFB1*	1.24	TGF-β Signaling (MIM:190180)
*TGFB2*	0.74	TGF-β Signaling (MIM:190220)
*TGFB3*	0.77	TGF-β Signaling (MIM:190230)
*TIMP1*	0.88	Extracellular Matrix Protease Inhibitor (MIM:305370)
*VCAN*	0.88	Cell Adhesion Molecule (MIM:118661)
*RPL13*	1.00	Housekeeping gene (MIM:113703)

The bold values represent the statistically significant results (fold change ≥ 2 and p-value ≤ 0.05 for upregulated genes, and fold change ≤ 0.5 and *p*-value ≤ 0.05 for significantly downregulated genes).

**Table 3:** Significant gene expression levels after 24 h of treatment with mycophenolate, as compared with untreated cells.

Gene	Fold Change	SD (+/−)	Gene Function
*CDH1*	4.62	0.00	Cell-Cell Adhesion
*COL1A2*	2.20	0.07	Collagens and Extracellular Matrix Structural constituent
*COL4A1*	3.43	0.15	Collagens and Extracellular Matrix Structural constituent
*COL8A1*	2.54	0.02	Collagens and Extracellular Matrix Structural constituent
*COL10A1*	2.10	0.00	Collagens and Extracellular Matrix Structural constituent
*CCTNA1*	2.19	0.07	Cell Adhesion Molecule
*CTNB*	2.36	0.06	Cell Adhesion Molecule
*ITGA1*	3.72	0.18	Transmembrane Receptor
*ITGA2*	5.30	0.16	Transmembrane Receptor
*ITGA3*	3.04	0.12	Transmembrane Receptor
*ITGA5*	2.82	0.15	Transmembrane Receptor
*ITGA7*	4.81	0.06	Transmembrane Receptor
*ITGB1*	2.22	0.29	Transmembrane Receptor
*LAMB2*	2.08	0.00	Basement Membrane Constituent
*LAMB3*	4.86	0.10	Basement Membrane Constituent
*MMP3*	2.90	0.02	Extracellular Matrix Protease
*MMP7*	2.37	0.48	Extracellular Matrix Protease
*MMP8*	0.45	0.00	Extracellular Matrix Protease
*MMP10*	4.59	0.17	Extracellular Matrix Protease
*MMP11*	0.03	0.00	Extracellular Matrix Protease
*MMP12*	4.26	0.02	Extracellular Matrix Protease
*MMP13*	3.82	0.26	Extracellular Matrix Protease
*MMP15*	0.06	0.00	Extracellular Matrix Protease
*MMP16*	0.42	0.02	Extracellular Matrix Protease
*MMP24*	0.09	0.02	Extracellular Matrix Protease
*MMP26*	3.43	0.39	Extracellular Matrix Protease

Fold change ≥ 2 and *p*-value ≤ 0.05 for upregulated genes, and fold change ≤ 0.5 and *p*-value ≤ 0.05 for significantly downregulated genes.

**Table 4:** Significant gene expression levels after 24 h of treatment with cyclosporine, as compared with untreated cells [34].

Gene	Fold Change	SD (+/−)	Gene Function
*CDH1*	5.01	0.17	Cell-Cell Adhesion
*COL7A1*	0.26	0.02	Collagens and Extracellular Matrix Structural constituent
*ITGA2*	2.05	0.07	Transmembrane Receptor
*ITGA7*	2.75	0.52	Transmembrane Receptor
*ITGB2*	0.34	0.12	Transmembrane Receptor
*ITGB4*	0.19	0.02	Transmembrane Receptor
*LAMA2*	0.15	0.02	Basement Membrane Constituent
*LAMB1*	0.06	0.00	Basement Membrane Constituent
*LAMB3*	2.37	0.14	Basement Membrane Constituent
*MMP8*	0.05	0.01	Extracellular Matrix Protease
*MMP11*	0.03	0.00	Extracellular Matrix Protease
*MMP12*	2.07	0.08	Extracellular Matrix Protease
*MMP13*	2.03	0.01	Extracellular Matrix Protease
*MMP15*	0.05	0.01	Extracellular Matrix Protease
*MMP16*	0.31	0.02	Extracellular Matrix Protease
*MMP24*	0.04	0.01	Extracellular Matrix Protease
*MMP26*	0.49	0.15	Extracellular Matrix Protease

Fold change ≥ 2 and *p*-value ≤ 0.05 for upregulated genes, and fold change ≤ 0.5 and *p*-value ≤ 0.05 for significantly downregulated genes.
